# Supplementary material for: miR-196a Upregulation Contributes to Gefitinib Resistance through Inhibiting GLTP Expression
Source: Int J Mol Sci. 2022 Feb 4;23(3):1785. doi: 10.3390/ijms23031785 (PMC8836598; doi:10.3390/ijms23031785)
Supplement: Supplementary file 1 [file ijms-23-01785-s001.zip › ijms-1431234-supplementary.pdf]

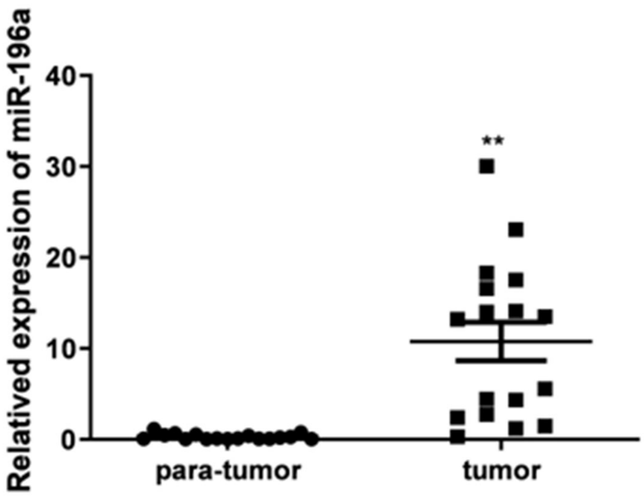


**Figure S1. The expression levels of miR-196a were much higher in LUAD.** The expression levels of miR-196a were examined in clinical lung tumor and adjacent tissues by qRT-PCR (para-tumor: n=17, tumor: n=17). Data represent the mean ± S.D. of 3 independent experiments where **indicates significant difference at $p$ <0.01.

**A**

| Matrix ID | Name | Score | Relative score | Sequence ID | Start | End | Strand | Predicted sequence |
|---|---|---|---|---|---|---|---|---|
| MA0150.1 | MA0150.1.NFE2L2 | 15.1208515 | 0.9775409770790805 | hg38_wgRna_hsa-mir-196a-1 | 329 | 339 | + | gtgacacagca |
| MA0150.1 | MA0150.1.NFE2L2 | 9.303911 | 0.8329571010621472 | hg38_wgRna_hsa-mir-196a-1 | 588 | 598 | - | ctgacccagcc |
| MA0150.1 | MA0150.1.NFE2L2 | 9.256913 | 0.8317889342744886 | hg38_wgRna_hsa-mir-196a-1 | 1689 | 1699 | + | gagactgagca |

**B**

| Matrix ID | Name | Score | Relative score | Sequence ID | Start | End | Strand | Predicted sequence |
|---|---|---|---|---|---|---|---|---|
| MA0095.1 | MA0095.1.YY1 | 7.219338 | 0.9405610215352127 | hg38_wgRna_hsa-mir-196a-1 | 1940 | 1945 | - | gccatt |
| MA0095.1 | MA0095.1.YY1 | 7.0274825 | 0.9307622883769914 | hg38_wgRna_hsa-mir-196a-1 | 1818 | 1823 | - | accatt |
| MA0095.2 | MA0095.2.YY1 | 13.80733 | 0.9252071359660693 | hg38_wgRna_hsa-mir-196a-1 | 396 | 407 | + | aaaaatggaggc |
| MA0095.2 | MA0095.2.YY1 | 13.514719 | 0.9209615693405562 | hg38_wgRna_hsa-mir-196a-1 | 1937 | 1948 | + | aaaaatggccac |
| MA0095.1 | MA0095.1.YY1 | 6.3996224 | 0.8986952613442417 | hg38_wgRna_hsa-mir-196a-1 | 1090 | 1095 | - | cccatc |
| MA0095.1 | MA0095.1.YY1 | 6.2248564 | 0.8897693423966765 | hg38_wgRna_hsa-mir-196a-1 | 399 | 404 | - | tccatt |
| MA0095.2 | MA0095.2.YY1 | 10.039752 | 0.8705424197720857 | hg38_wgRna_hsa-mir-196a-1 | 1815 | 1826 | + | caaaatggtttt |
| MA0095.1 | MA0095.1.YY1 | 5.23583 | 0.8392562702106197 | hg38_wgRna_hsa-mir-196a-1 | 1388 | 1393 | + | cccatt |
| MA0095.2 | MA0095.2.YY1 | 5.313941 | 0.8019744674207386 | hg38_wgRna_hsa-mir-196a-1 | 1323 | 1334 | - | caaaatgtccca |

**C**

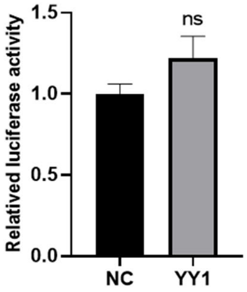


**Supplemental Figure S2. NRF2, but not YY1 regulated the expression levels of miR-196a at transcriptional level. (A)** The sites and scores of NRF2 binding miR-196a promoter regions were predicted by Jasper website. **(B)** The sites and scores of YY1 binding miR-196a promoter region were predicted by Jasper website. **(C)** The 2kb upstream sequence of miR-196a transcription start site was cloned into PGL3 vector, which co-transfected with YY1 overexpression vector in 293T cells. The potential YY1 binding regions in miR-196a promoter were tested by luciferase report assay. Data represent the mean ± S.D. of 3 independent experiments where ns stands for no significant difference.

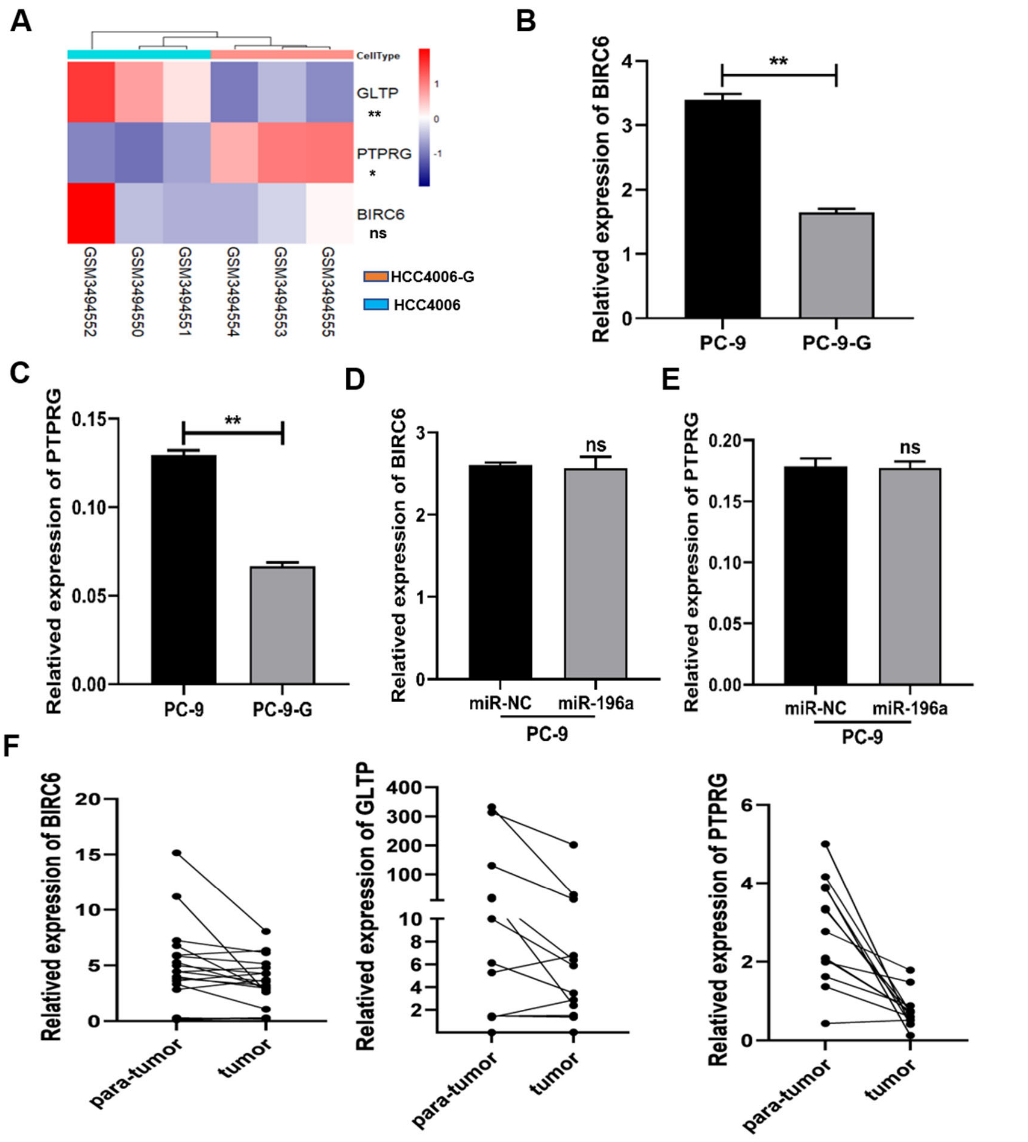


**Supplemental Figure S3. GLTP was the top potential target of miR-196a. (A)** Predicted direct target genes of miR-196a were analyzed in parental HCC4006 and corresponding gefitinib resistant cells by GEO databases. GLTP was the top potential target, which was further confirmed as direct target of miR-196a (Figure 4). **(B-C)** The mRNA expression levels of two candidate genes were measured in PC-9 and corresponding resistant PC-9-G cells. **(D-E)** Forced expression of miR-196a did not decrease mRNA expression levels of BIRC6 and PTPRG. The mRNA expression levels of BIRC6 and PTPRG in miR-196a overexpression cells were detected using qRT-PCR. **(F)** The mRNA expression levels of three predicted genes were tested in lung cancer

tissues and adjacent tissues. Data represent the mean ± S.D. of 3 independent experiments where ** indicates significant difference at $p$ <0.01, ns indicates no significant difference.

**Table S1:**

| | Sequence (5'→3') | |
|---|---|---|
| genes | Forward | Reverse |
| TBP | CACGAACCACGGCACTGATT | TTTTCTTGCTGCCAGTCTGGAC |
| GLTP | AAGCTGTGTACGACACCAACC | GCGTTGACACGGATGAGGT |
| PTPRG | TGACAGAAGGCTACGTTGGG | TCAGGACCATAGGCACCAGAG |
| BIRC6 | TAGTGTATGCCTCGTTTGTTGG | TTCTGTGTGTGCTCACCTTTC |

**Table S1. The primer sequences used in qRT-PCR.**
